# The DLO Hi-C Tool for Digestion-Ligation-Only Hi-C Chromosome Conformation Capture Data Analysis

**DOI:** 10.3390/genes11030289

**Published:** 2020-03-10

**Authors:** Ping Hong, Hao Jiang, Weize Xu, Da Lin, Qian Xu, Gang Cao, Guoliang Li

**Affiliations:** 1 National Key Laboratory of Crop Genetic Improvement, Huazhong Agricultural University, Wuhan 430070, China; hongping@webmail.hzau.edu.cn (P.H.); jianghao94@webmail.hzau.edu.cn (H.J.); 2014305201215@webmail.hzau.edu.cn (Q.X.); 2 Agricultural Bioinformatics Key Laboratory of Hubei Province, Hubei Engineering Technology Research Center of Agricultural Big Data, College of Informatics, Huazhong Agricultural University, Wuhan 430070, China; 3 State Key Laboratory of Agricultural Microbiology, Huazhong Agricultural University, Wuhan 430070, China; vet.xwz@webmail.hzau.edu.cn (W.X.); dalin@mail.hzau.edu.cn (D.L.); 4 College of Veterinary Medicine, Huazhong Agricultural University, Wuhan 430070, China; 5 College of Bio-Medical and Health, Huazhong Agricultural University, Wuhan 430070, China

**Keywords:** 3D genomics, DLO Hi-C, linker detection, iteration mapping

## Abstract

It is becoming increasingly important to understand the mechanism of regulatory elements on target genes in long-range genomic distance. 3C (chromosome conformation capture) and its derived methods are now widely applied to investigate three-dimensional (3D) genome organizations and gene regulation. Digestion-ligation-only Hi-C (DLO Hi-C) is a new technology with high efficiency and cost-effectiveness for whole-genome chromosome conformation capture. Here, we introduce the DLO Hi-C tool, a flexible and versatile pipeline for processing DLO Hi-C data from raw sequencing reads to normalized contact maps and for providing quality controls for different steps. It includes more efficient iterative mapping and linker filtering. We applied the DLO Hi-C tool to different DLO Hi-C datasets and demonstrated its ability in processing large data with multithreading. The DLO Hi-C tool is suitable for processing DLO Hi-C and in situ DLO Hi-C datasets. It is convenient and efficient for DLO Hi-C data processing.

## 1. Introduction

Recently, there is increasing evidence that higher-order chromatin structures play an important role in gene expression and regulation [1]. In order to dissect the three-dimensional (3D) structure of the chromatin, the chromosome conformation capture (3C) method [2], which depends on DNA proximity ligation [3], was first proposed in 2002. With the rapid progression of high-throughput sequencing technology and bioinformatics, various derivative methods such as Hi-C [4], ChIA-PET (Chromatin Interaction Analysis by Paired-End Tag Sequencing) [5], DNase Hi-C [6], in situ Hi-C [7], and capture Hi-C [8] were developed to study the 3D genome organization and chromosomal interactions. Hi-C is a powerful method to investigate the genome-wide all-to-all long-range chromatin interactions and topologically associating domains (TADs) [9] of the genome, and it has contributed to great achievements in understanding the principle of biological functions from the perspective of the 3D chromosomal structure.

Although Hi-C has greatly advanced our understanding of the 3D organization of genomes, it has some limitations, such as a high rate of randomly ligated DNA noise, a high cost, and complex experimental procedures. To overcome the limitations of the pre-existing Hi-C method, we developed a simple, cost-effective, and low-noise-level method: digestion-ligation-only Hi-C (DLO Hi-C) [10]. The key advantages of DLO Hi-C are as follows. Firstly, the main steps of the DLO Hi-C method are simplified, with only two rounds of digestion and ligation. Secondly, it is convenient to estimate the signal-to-noise ratio from different combinations of linkers, which is not available for other Hi-C methods. Thirdly, the linker-ligated fragments are purified by cost-effective poly acrylamide gel electrophoresis (PAGE) instead of biotin labeling and pulling down steps, which reduces the cost of the experiment. Fourthly, simultaneous digestion and ligation steps can effectively reduce the percentage of re-ligation reads (the paired-end DNA interaction information derived from two adjacent DNA fragments). Lastly, because of the delicate design of specific nucleotide barcodes, double digestion is convenient for use as an early quality-control step for examining the ratios of ligation compositions before sequencing to evaluate the quality of the DLO Hi-C library. By applying two rounds of digestion and ligation, approximately 80-bp DLO Hi-C DNA fragments are generated, which contains a 40-bp complete linker and a pair of 20-bp interacting genomic DNA fragments. These DLO Hi-C fragments are subjected to high-throughput sequencing. A pair of the mapped genomic DNA fragments will uncover chromatin interactions, and self-ligation products from the two ends of the individual DNA fragments or re-ligation products take just a small proportion in the uniquely mapped reads.

Originally, we combined some components from ChIA-PET and Hi-C data analysis packages and shell scripts for DLO Hi-C data analysis, since the DLO Hi-C protocol included different experimental steps from ChIA-PET and Hi-C methods. The ChIA-PET tool [11] was the first package designed for ChIA-PET data processing. Since the DNA construct of DLO Hi-C data is similar to that of the short reads in ChIA-PET data, the linker reads were recognized and filtered with a modified ChIA-PET tool [10]. However, the following steps in the ChIA-PET tool are not suitable for DLO Hi-C data due to different noise sources. HiC-Pro [12] is the commonly used pipeline for Hi-C data processing. Since the construct of DLO Hi-C data is different from that of traditional Hi-C data, the traditional Hi-C pipeline is not available for DLO Hi-C data [10]. Due to lacking the linker filtering process, HiC-Pro is incapable of processing DLO Hi-C data. Besides, the noise filtering is different from traditional methods. Some in-house shell scripts were first developed to process the DLO Hi-C data, but it was complicated and not friendly for users. As far as we know, there is no public pipeline appropriate for DLO Hi-C data. In order to facilitate the analysis of DLO Hi-C data, here we present an easy-to-use and versatile pipeline called the “DLO Hi-C tool” to process DLO Hi-C data from raw sequencing reads to normalized contact maps.

In this study, we provide a detailed description of the design and implementation of the DLO Hi-C tool. By applying the DLO Hi-C tool to different DLO Hi-C datasets, we demonstrate its efficiency and effectiveness. Quality control at different steps of the DLO Hi-C analysis is supported in our pipeline and will be displayed in the final report files.

## 2. Materials and Methods

### 2.1. Data Accessibility

The dataset used in this project was downloaded from the National Center for Biotechnology Information (NCBI) Gene Expression Omnibus (GEO) dataset with accession number GSE89663. The human reference genome hg19 was retrieved from the ENSEMBL website (ftp://ftp.ensembl.org/pub/release-73/fasta/homo_sapiens/dna/).

### 2.2. Package Availability and Requirements

Project name: DLO Hi-C Tool.

Project home page: https://github.com/GuoliangLi-HZAU/DLO-Hi-C-Tool (Java version) and https://github.com/GangCaoLab/DLO-HiC-Tools (Python version).

Operating system(s): Linux.

Programming language: Java.

Other software dependencies: JRE 1.8 or higher; mafft; BWA; Python 2.7; Python package: Matplotlib, SciPy, argparse, and NumPy ≤ 1.14.2.

License: GPL license.

Any restrictions to use by nonacademics: license needed.

### 2.3. Design and Implementation

**Figure 1:**
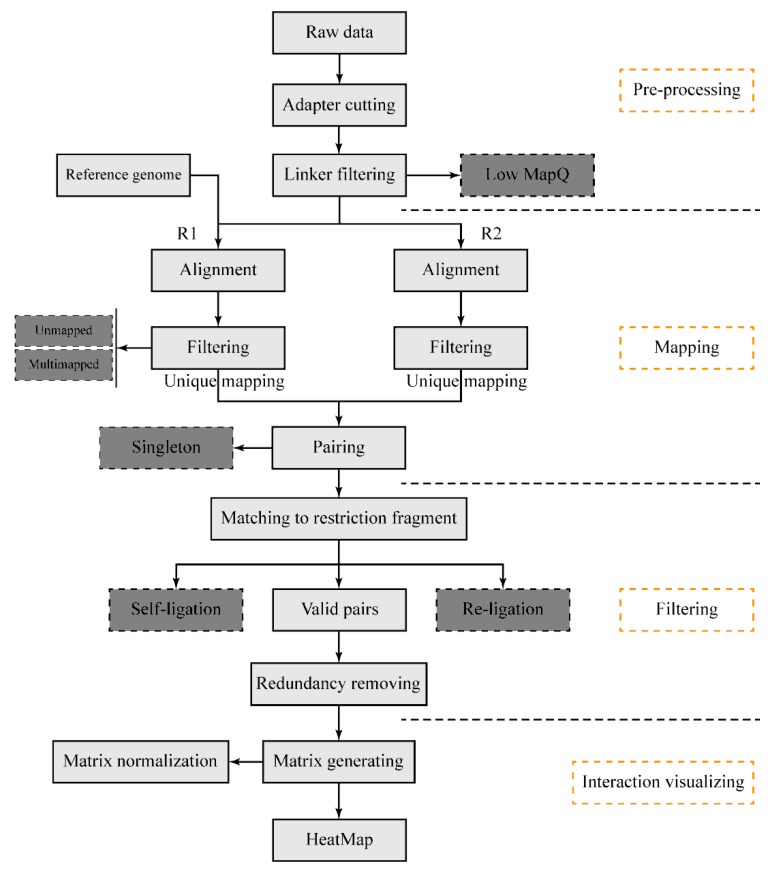
The schematic pipeline for the digestion-ligation-only Hi-C (DLO Hi-C) tool. The entire DLO Hi-C tool pipeline consists of four main steps, as four separate modules to process DLO Hi-C data. Boxes in dark gray indicate the filtered-out reads.

The DLO Hi-C tool pipeline (Figure 1) begins with sequencing reads from the DLO Hi-C library. There are four main steps: pre-processing of raw sequencing reads, reads alignment and filtering, noise reduction and paired-end reads classification, and interaction visualization. It is available for users to flexibly modify any modules. Information can be transported through file systems between steps, so that users can resume the process if the program is interrupted by accident. This pipeline is written in the Java programming language and compiled in the jar package. There is no specific installation process required for the program, but users need to ensure their Java version is no less than 1.8 and the dependencies have been correctly installed in the virtual environment.

### 2.4. Configuration File

There is only one configuration file in the DLO Hi-C tool, and the content is divided into three sections: required parameters, optional parameters, and advanced parameters. The required parameters need to be modified for each DLO Hi-C library, but the optional and advanced parameters can be kept as the default values (advanced parameter need not be modified in common). The format of the configuration file contains three parts: the name of the argument, the equal sign, and the value of the parameters. Users can set the value of the optional parameters as empty if they want to use a default value or have no idea of the specific information. The program can generate a template file if the user does not know about the content of the configuration file.

We have also provided a graphical user interface for users to conveniently set the parameters.

### 2.5. Data Pre-Processing

#### 2.5.1. Adapter Cutting

**Figure 2:**
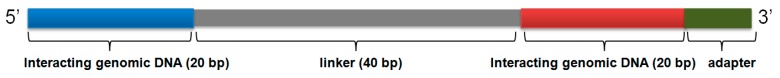
The composition of a DLO Hi-C DNA fragment. The DLO Hi-C DNA fragment contains two interacting genomic DNAs, 40 bp linker sequences, and adapter bases.

The effective sequence from the DLO Hi-C construct is about 80 bp (Figure 2). With the present sequencing capacity, single-end sequencing can obtain the full-length of the DNA constructs. Usually, the length of paired-end sequencing reads is longer than 80 bp. If the paired-end sequencing protocol is applied, only the first sequenced reads, which contain the whole effective sequences, are kept for further analysis. In the current sequencing protocol, indexed adapters are generally added at the end of the reads (Figure 2). At the beginning of the data processing, the adapters need to be clipped from the raw reads (Figure 1). The input file can be in FASTQ or compressed FASTQ format. In order to obtain accurate sequences of interactions, the accuracy of the start position of the adapter is very critical. At this step, mafft [13] is used to recognize the sequences of the adapter. By default, multiple sequence alignment is conducted on 100 reads, and the adapter sequence is recognized based on the alignment results. The program counts the base frequency at each position from the alignment results and then estimates the most probable bases at each position. Then, the adapter sequences are detected and cut from the raw reads. Adapter cutting is quite a flexible feature of this program; users can either specify the adapter sequences in the “AdapterSeq” parameter or can keep it blank if adapter cutting is not required.

#### 2.5.2. Linker Detection and Filtering

Due to the specific linker sequences, it is easy to distinguish the interacting fragments from genomic DNA sequencing reads. Besides this, the linkers are also designed to estimate the ratio of random ligation. The convenience to estimate the random ligation noise with linkers contributes to the advantages of the DLO Hi-C method. The DLO Hi-C tool makes a combination of all possible linkers according to the input half-linker sequences.

There are four combinations (AA, BB, AB, and BA) of half-linkers in DLO Hi-C and one combination (AA) of half-linkers in the in situ DLO Hi-C library. The length of the linkers is about 40 bp, which is located in the middle of the sequenced reads. The combinations of linkers (two half-linkers) are first aligned to the adapter clipped reads to get the precise position of the linkers (Figure 1). The result file of this step contains the information of every read, such as the DNA sequence in the left side of the linker, the start index of the linker, the end index of the linker, the DNA sequence in the right side of the linker, the start position of the adapter, the linker type, the mapping quality, the read label, the read sequence, the sequence orientation, and the sequencing quality. Then, the reads with linkers are separated into four different files (for DLO Hi-C data) or one file (for in situ DLO Hi-C data) based on linker types for the subsequent steps.

When the prior information of the linker sequence is missing, it is essential to detect it automatically. With this consideration, a module was designed and incorporated into this pipeline to recognize the linker sequence from the DLO Hi-C dataset. This module can detect not only the linker sequence but also the enzyme sites used in the experiment. To the best of our knowledge, it is the first pipeline that can detect linker sequences and enzyme cutting sites automatically. Without the information of the linker or the enzyme, users can set the “HalfLinker” or “Restriction” value to an empty string in the pipeline.

### 2.6. Mapping

#### 2.6.1. Mapping the Reads to the Reference Genome

**Figure 3:**
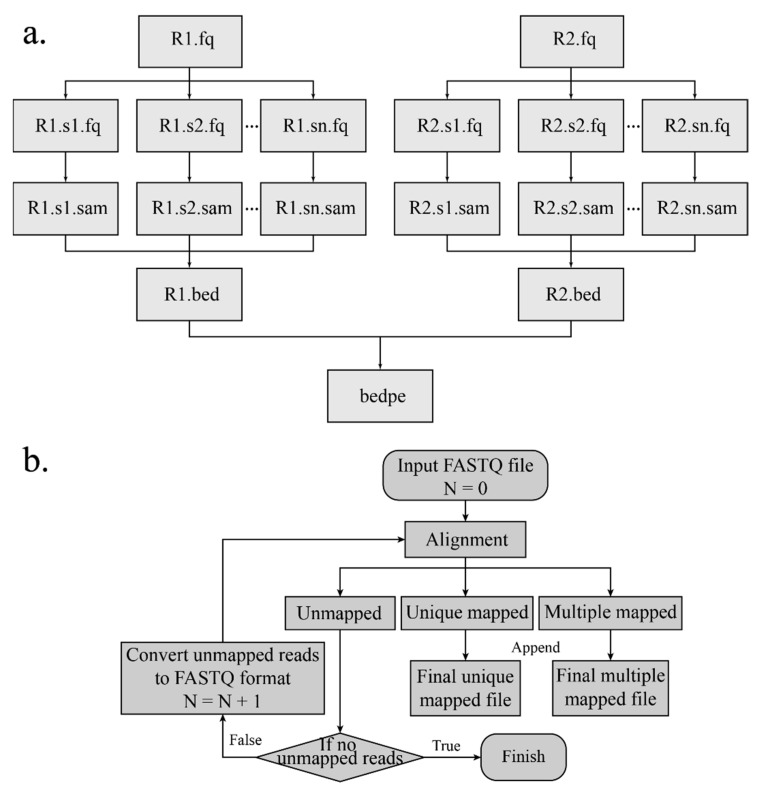
Flow chart of mapping and iterative mapping. (**a**) Raw data are split into several chunks, and the alignment results of different chunks are merged after pairing. (**b**) The flowchart shows the detailed steps of iterative mapping. N represents the maximum edit distance of the Burrows-Wheeler Alignment (BWA).

The interaction products can be obtained according to the start and end indexes of the linkers. Read pairs are aligned to the corresponding reference genome separately by Burrows-Wheeler Alignment (BWA) [14,15] with an aln algorithm. The SAI file is produced with 0 maximum edit distance and other default arguments. The conversion of the SAI file is very time-consuming and cannot be processed in a parallel mode. In order to make alignment time efficient, the FASTQ file is split into several chunks, and the alignment of each chunk is conducted on each individual thread. After aligning to the reference genome and converting (Figure 3a), the mapping results are merged. Besides, if the sequence reads are long (more than 70 bp), the alignment of the long sequence is supported by setting “ReadsType” as “long” for the mem algorithm of the BWA. Based on the mapping score, reads are divided into three categories: Uniquely mapping reads with a mapping score over 20, multi-mapped reads with a mapping score below 20, and unmapped reads with a mapping score of 0. If the MEM algorithm of the BWA is applied for alignment, the mapping score cutoff is set to 30.

#### 2.6.2. Iterative Mapping

The DLO Hi-C tool supports an additional mapping method named “iterative mapping”, and users can choose whether to use this method when mapping. There is a parameter “n” in the BWA aln algorithm, and the value represents the maximum mismatch base allowed. In the beginning (Step 1), the maximum edit distance is set to 0 (Figure 3b). After mapping, all reads are classified into three categories—mapped, unmapped, and multi-mapped. In Step 2, the reads corresponding to the unmapped category in the previous step undertake alignment again with “n” = 1, and the alignment results are classified into three categories. In Step 3, the reads in the unmapped category from Step 2 are mapped with one more mismatch allowed, which is repeated with iterative mapping until no unmapped reads remain. At the end, all reads of the “mapped” and “unmapped” categories are retained and merged. Iterative mapping can utilize the reads, which include SNP (single nucleotide polymorphism) or sequencing error, so that it can increase and improve the unique mapping ratio and the data utilization rate, respectively.

### 2.7. Noise Reduction and Paired-End Reads Classification

#### 2.7.1. Classification of Paired-End Reads Based on Alignment

**Figure 4:**
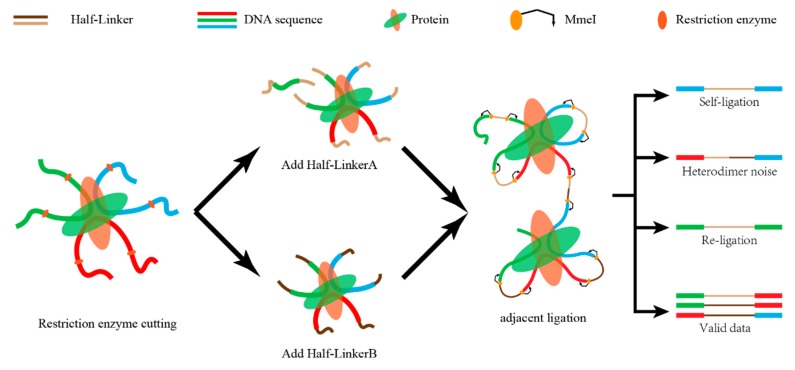
A sketch map of self-ligation and re-ligation. The fragments of interacting DNA pairs are divided into four categories—self-ligation, heterodimer noise, re-ligation, and valid data. Only the valid data are kept for further analysis.

Uniquely aligned reads at each end are paired with each other based on their sequence number. During this process, unpaired sequences (singleton) are discarded. Some of the paired-end reads are invalid interactions, such as re-ligation and self-ligation reads [10]. If the two ends of the reads correspond to two adjacent restriction fragments, the reads are called re-ligation reads. These reads are ligated with the same linker after digestion. If the two ends of a DNA fragment are ligated with one linker after digestion, the corresponding reads are self-ligation ends (Figure 4). In addition, there are some duplicate reads produced by polymerase chain reaction (PCR). The combination of different linkers is used to estimate the ratio of random collision which cannot be obtained from the real biological complexes.

#### 2.7.2. Duplication Removing

Duplicates with the same strands and positions of the two ends produced by PCR should be filtered out before interaction calling. Too many duplicates indicate high duplication noise. In order to reduce the effect of duplicate sequencing, we consider that reads with both ends mapped within a 2-bp difference, respectively, are duplicate reads. Then, we retain only one read of these reads for further analysis.

### 2.8. Interaction Matrix Converting and Normalization

The resolution is the bin size used to build the interaction matrices. The length of the restriction fragment decides the extreme resolution of the Hi-C library. The details of the interaction patterns differ for each resolution. We can get more information from a high-resolution (small bin size) map, but it needs more sequencing depth and is time-consuming in computation. The processing time is shorter for low-resolution (big bin size) maps, but some details may be lost. A balance is needed between the computing resources and the useful details. Users can define the values of the resolution in the configuration file to construct the interaction heatmaps. In order to offset the impact of distance, the program generates corresponding O/E (observed/expected) matrix [4] for each intra-chromosome interaction matrix.

### 2.9. Statistics Reports

The DLO Hi-C tool generates a HyperText Markup Language (HTML) report with all statistical results, including the parameters used in the pipeline, the information of the adapter and the linker sequences, and the running time of all steps.

### 2.10. Cross Validation

In order to evaluate the results of the DLO Hi-C tool, we used Pearson’s correlation coefficient (PCC) and cosine similarity to calculate the similarity between the results of the DLO Hi-C tool and the shell script. Cosine similarity is a method to calculate the distance of two vectors. If we have vector a and b, we know that a⋅b=||a||||b||cosθ, then:

$$\mathrm{Cosine}\;\mathrm{similarity}=cos\left(\theta \right)=\;\frac{A\cdot B}{\left||A|\right|\left||B|\right|}=\;\frac{\sum_{i=1}^{n}A_{i}\times B_{i}}{\sqrt{\sum_{i=1}^{n}{\left(A_{i}\right)}^{2}}\times \sqrt{\sum_{i=1}^{n}{\left(B_{i}\right)}^{2}}}$$

We needed to convert the interaction matrix to vectors, and the method of conversion is as follows:

$$\left[\begin{array}{ccc} a_{1,1} & \cdots & a_{1,n}\\ \vdots & \ddots & \vdots \\ a_{m,1} & \cdots & a_{m,n} \end{array}\right]\to \left|\begin{array}{c} row_{1}\\ \cdots \\ row_{m} \end{array}\right|\to \left|\begin{array}{ccc} row_{1} & \cdots & row_{m} \end{array}\right|$$

We combined all of the matrix items into one row to generate a vector and then used this vector to calculate the cosine similarity.

## 3. Results

The DLO Hi-C tool was used to detect the linker of a previously published DLO Hi-C dataset [10]. The test data included one DLO Hi-C library of the K562 cell line, three replicates of the in situ DLO Hi-C library of the K562 cell line, and two replicates of the in situ DLO Hi-C library of the THP-1 cell line. The paired-end reads length of the test datasets was 2 × 150 bp. Taking into account the quality of sequencing, we only kept the R1 end for subsequent analysis. The disk space of the test files is listed in Table 1.

### 3.1. Pre-Processing

Pre-processing includes five main steps: adapter detection, linker detection, linker alignment, separation, and linker filtering.

Table 2 shows the results of adapter and linker detection by the DLO Hi-C tool. The types of restriction enzymes and linker sequences were identical to the ones used in the experiments.

We used the algorithm of linker mapping in the ChIA-PET tool [11] as a reference and made some modifications to enable multi-thread execution. The parameter used in the alignment process is as follows: MatchScore: 1, MisMatchScore: −1, and InDelScore: −1. The linkers can be aligned to 10 million reads by the DLO Hi-C tool in 1 min and 42 s with 16 threads, which is much more efficient than the shell script pipeline (Table 3). The results of the preprocessing were saved in the 01. Preprocess directory, and it includes two FASTQ files and one txt file.

**Figure 5:**
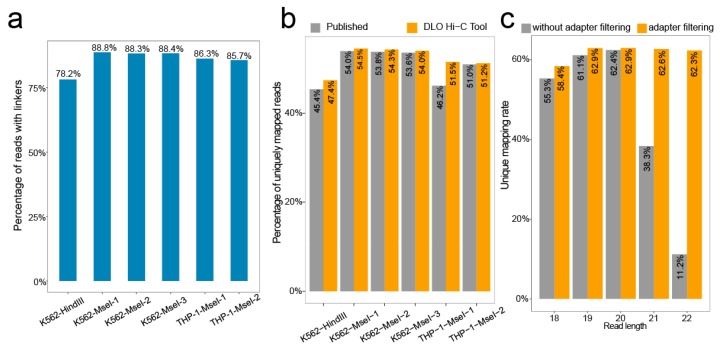
The ratio of linker sequence and unique mapping rate. (**a**) The proportion of reads containing the linker to the raw reads. (**b**) The uniquely mapping rate obtained from the DLO Hi-C Tool without iterative alignment and published results of different samples. (**c**) As the maximum read length increases, the unique mapping rate changes for performing adapter filtering or no adapter filtering.

We calculated the rate of the proportion of the sequences with linkers. Of all raw reads, the ratio of the linker sequence ranges from 78.2–88.8% (Figure 5a).

### 3.2. Alignment

BWA [14,15] was used to align the linker-filtered reads to the reference genome, and the default parameter was −t 1 −n 0; 20 was set as a cutoff score for unique mapping. The unique mapping ratio was similar to previously reported (Figure 5b), with the ratio ranging from 47.4–54.5%.

In addition, we tested the effect of adapter filtering on unique mapping by setting different maximum reads length (Figure 5c). By comparing the percentages of unique mapping with or without an adapter at different maximum cutting lengths, we found that the percentages of unique mapping were nearly the same when the cutting length was shorter than 20 bp. However, the percentages of unique mapping without adapter filtering declined rapidly with increasing cutting length for reads when the cutting length was longer than 20 bp. Overall, the percentages of unique mapping with adapter filtering were relatively stable.

The DLO Hi-C tool was used to process the DLO Hi-C and in situ DLO Hi-C datasets published by Lin et al. [10]. We compared the number of reads at every step. The numbers of reads were nearly the same between the two pipelines at every step. The unique mapping rate and data utilization rate had a slight improvement on the DLO Hi-C tool. Compared with the published results, the uniquely mapping rate and data utilization rate of iterative alignment improved by 7.4% and 5.9%, respectively (Table 4).

**Figure 6:**
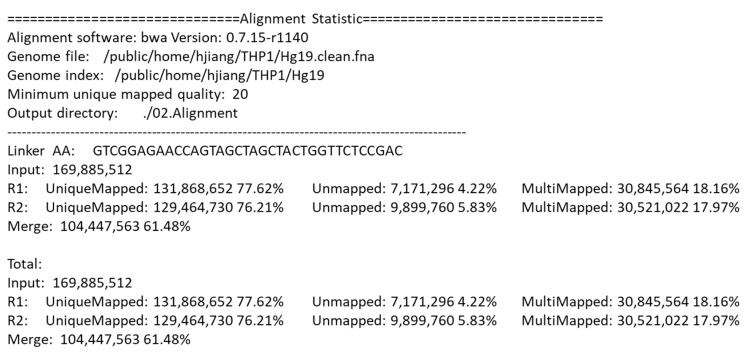
Statistics report of the alignment.

After alignment, the DLO Hi-C tool gives a statistical report. Figure 6 shows part of the alignment statistics in the final report. Besides the version of BWA and the directory of the genome file, the report also gives detailed information of the parameters and the results of the alignment, which is convenient for users to compare the results with different parameters.

### 3.3. Duplicate Filtering

**Figure 7:**
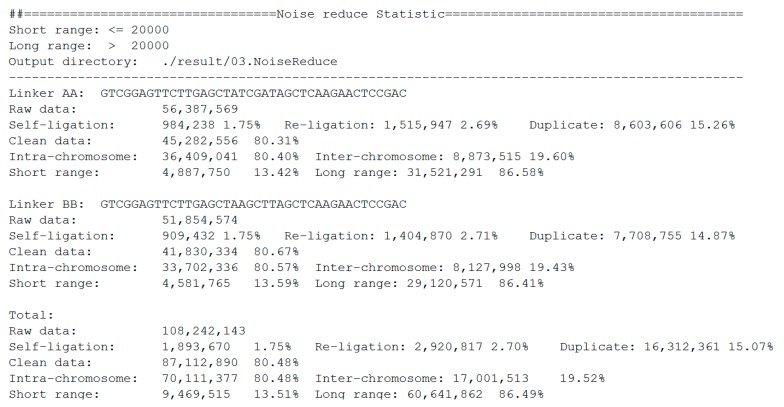
Statistics report of noise redundancy.

The statistics of noise redundancy are included in the final report (Figure 7). For linker AA and linker BB sequences, it shows the detailed numbers and percentages of self-ligation, re-ligation, duplication, intra-chromosome, inter-chromosome, short-range, and long-range reads separately. This report makes it easier for users to estimate the quality of the DLO Hi-C library.

### 3.4. Cross Validation

**Figure 8:**
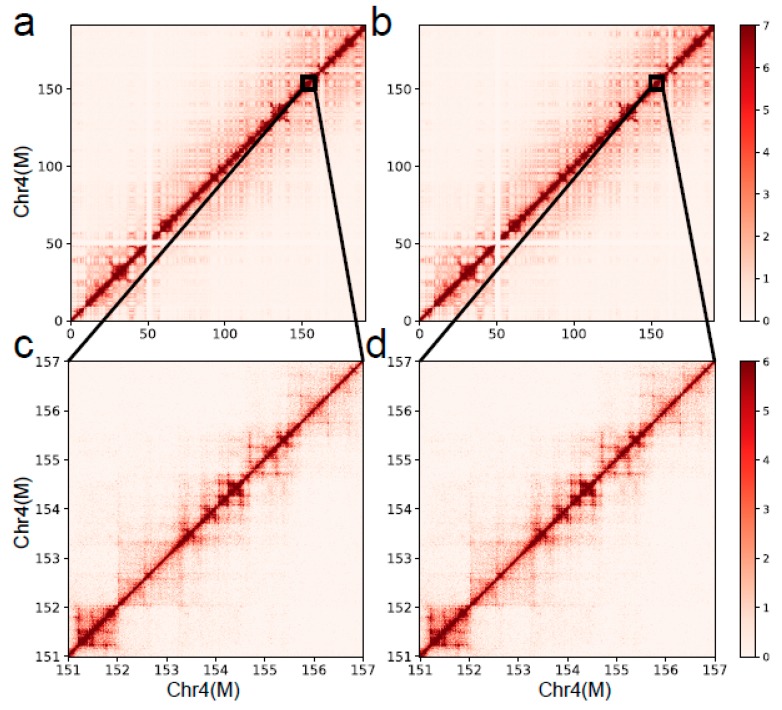
The interaction heatmaps of chromosome 4 from the K562 cell line. (**a** **and c**) The heatmaps of the whole and local chromosome 4 of K562 generated by the published results. (**b** **and d**) The interaction heatmaps of chromosome 4 and the enlarged region generated by the DLO Hi-C tool.

Cosine similarity and Pearson’s correlation coefficient (PCC) were applied to calculate the similarity between the results from the DLO Hi-C tool and the shell script. We generated a whole-genomic interaction matrix of the K562 cell line in 1-Mb resolution and calculated the similarity of the matrices using the DLO Hi-C tool and from the original article. The PCC and cosine similarity were both larger than 0.99 in K562-MseI-1 and K562-MseI-2, respectively, which indicates that the results of the DLO Hi-C tool have high similarity with the published results. Besides, the PCC and cosine similarity of each chromosome were larger than 0.99, which indicates the high similarity of the results (Supplementary 1: Table S1). We also compared the results of the DLO Hi-C tool with the published results through the interaction heatmap. Figure 8 shows the chromatin-interaction heatmaps obtained by the DLO Hi-C tool and the published results. We can see a high consistency of chromosome 4 from the heatmaps.

**Figure 9:**
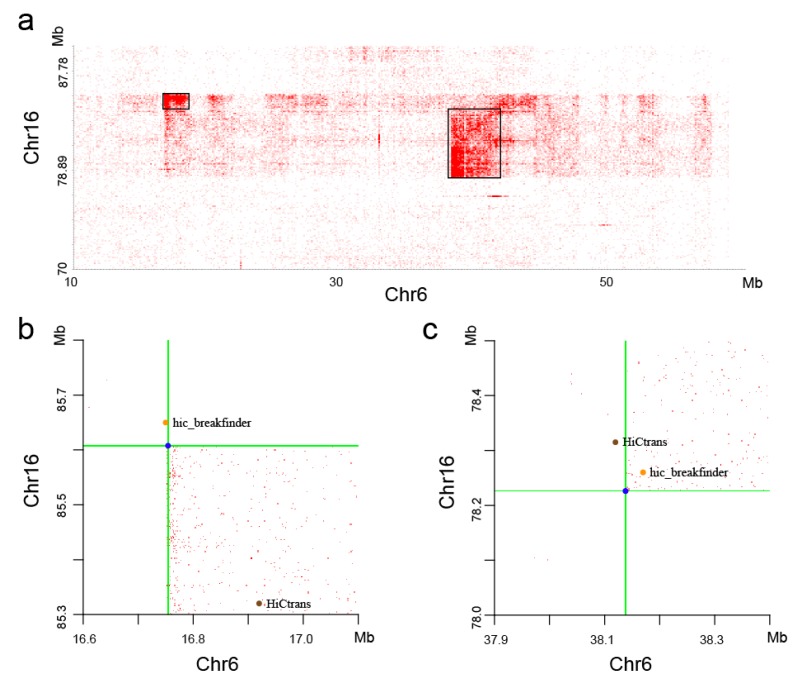
Translocation regions between chr6 and chr16. (**a**) The translocation region obtained from the heatmaps generated by the DLO Hi-C tool, with the translocations marked as black rectangles. (**b and c**) The translocations found by hic_breakfinder and HiCtrans.

With visual observation of the heatmaps, we found some potential chromosome translocations, as shown in Figure 9. We confirmed the authenticity of these two translocations by the software hic_breakfinder [16] and HiCtrans [17]. The heatmap generated by the DLO Hi-C tool provides a visual basis for chromatin translocation calling.

### 3.5. HTML Report

In the final HTML report, users can visually see the specific information of the entire DLO Hi-C library (Supplementary 2, Supplementary 3). The report mainly shows five aspects: running information, linker filtering, alignment, noise reduction, and interaction matrix report.

The running information table indicates the detailed parameters used in the program. The linker filtering section contains basic statistics, base distribution in adapter detection, linker alignment score distribution, and tag length distribution. From this part, it is easy to determine the percentage and mapping details of the linker sequences. The basic statistics of the alignment provide the information of the reads number and percentages of different categories. In the basic statistics of the noise reducing section, we can obtain the number and proportion of the sequences of noise from different sources. Additionally, the number and proportion of long- and short-range interactions are listed in the table. Furthermore, the statistics of the positive and negative strands and the interaction distribution are concluded in the noise-reducing part. The interaction matrix report part displays the interaction heatmap of the whole genome.

## 4. Conclusions and Discussion

The DNA sequence construct of the DLO Hi-C library is different from that of the traditional Hi-C library. Based on the characteristics of the DLO Hi-C library, there are two main steps: preprocessing and mapping, which cannot be performed by already publicly available Hi-C pipelines.

Compared with the traditional Hi-C library, the length of interacting DNA fragments in the DLO Hi-C library is much shorter. For the alignment of short reads, the length of the reads has a great impact on the unique mapping rate. Therefore, accurately locating the linker and adapter position is very important to improve data utilization.

In this paper, we developed the DLO Hi-C tool for DLO Hi-C data analysis. In addition to the Java version, we also developed the Python version pipeline (Supplementary 4). By processing the data from the article of the DLO Hi-C experiment, we demonstrated that the DLO Hi-C tool can achieve results similar to those from the original shell scripts, and it is more efficient. We compared the features of DLO Hi-C tool with the original shell scripts and found that the DLO Hi-C tool has more advantages than the shell script (Table 5). This tool supports FASTQ and compressed FASTQ files as input and has less software dependencies. It requires less input information, so that it is easier to use than the shell script. It can process data from other species without modifying any code. The DLO Hi-C tool consumes much memory when many threads are used; therefore, the number of threads used needs to be set as a small number, if the memory is not enough. We found that the position of the reads mapped in the enzyme fragment has a strong correlation with the forward and reverse strands (Table 6). This result conforms to the theory of the DLO Hi-C experiment, but we can see that there are some abnormal values with non-expected combinations of the strand and end distributions. We guess that such reads could be from noise or random ligation, which needs further exploration in the future.

## Figures and Tables

**Table 1:** The disk space of the test files.

Cell Line	Enzyme	Dataset	Raw Reads	File Size
K562	*Hind*III	K562-HindIII	228,257,962	77 G
K562	*Mse*I	K562-MseI-1	191,931,665	64 G
K562	*Mse*I	K562-MseI-2	208,310,948	70 G
K562	*Mse*I	K562-MseI-3	262,150,931	88 G
THP-1	*Mse*I	THP-1-MseI-1	402,394,454	145 G
THP-1	*Mse*I	THP-1-MseI-2	431,193,228	156 G

The first column contains the cell line types of the data, the second column corresponds to the restriction enzyme used in the experiment, the third column displays the labels of the datasets, the fourth column shows the number of raw reads in the Read1 file, and the fifth column indicates the disk space of the raw data in the Read1 file with FASTQ format.

**Table 2:** Digestion-ligation-only (DLO) Hi-C tool performance on enzyme and linker detection.

Dataset		Restriction	Half-Linker A	Half-Linker B
K562-HindIII	Real	A^AGCTT	GTCGGAGTTCTTGAGCTAAG	GTCGGAGTTCTTGAGCTATC
	Detected	A^AGCTT	GTCGGAGTTCTTGAGCTAAG	GTCGGAGTTCTTGAGCTATC
K562-MseI-1	Real	T^TAA	GTCGGAGAACCAGTAGCT	
	Detected	T^TAA	GTCGGAGAACCAGTAGCT	
K562-MseI-2	Real	T^TAA	GTCGGAGAACCAGTAGCT	
	Detected	T^TAA	GTCGGAGAACCAGTAGCT	
K562-MseI-3	Real	T^TAA	GTCGGAGAACCAGTAGCT	
	Detected	T^TAA	GTCGGAGAACCAGTAGCT	
THP-1-MseI-1	Real	T^TAA	GTCGGAGAACCAGTAGCT	
	Detected	T^TAA	GTCGGAGAACCAGTAGCT	
THP-1-MseI-2	Real	T^TAA	GTCGGAGAACCAGTAGCT	
	Detected	T^TAA	GTCGGAGAACCAGTAGCT	

**Table 3:** Running time of linker filtering (K562-MseI-1).

	Thread	4	8	12	16	Shell
Reads						
10 million		3 m 57 s	2 m 21 s	1 m 49 s	1 m 42 s	10 m 13 s
20 million		7 m 51 s	4 m 43 s	3 m 38 s	3 m 25 s	22 m 36 s
40 million		15 m 44 s	9 m 26 s	7 m 21 s	6 m 52 s	59 m 12s

**Table 4:** Comparison of different strategies.

	Lin et al. [10]	Without Iterative Mapping	With Iterative Mapping
Dataset	K562-MseI-1	K562-MseI-1	K562-MseI-1
Digestion enzyme	*Mse*I	*Mse*I	*Mse*I
Raw reads	191,931,665	191,931,665	191,931,665
Linker reads	169,854,725	169,885,029	169,885,029
Uniquely mapped reads	103,549,004	104,506,001	111,270,455
Nonredundant mapped reads	96,795,788	96,328,637	102,462,556
Inter-chromosomal contacts	17,637,527	16,542,652	18,180,661
Intra-chromosomal contacts	79,158,261	79,785,985	84,281,895
Intra-short-range contacts ≤ 5K	18,740,658	18,806,726	19,840,760
Intra-long-range contacts > 5K	60,417,603	60,979,259	64,441,135

**Table 5:** Comparison of the DLO Hi-C tool with the original shell script.

Item	Shell Script	DLO Hi-C Tool
Input file format	FASTQ	Original or gz compressed FASTQ
Thread	Single	Multiple
Software dependence	BWA, SAMtools, perl, BEDTools, Python	BWA, mafft, python
User-friendliness	Moderate	Good
Resume from break-point	Not supported	Supported
HTML report	No	Yes
Linker sequence	Required	Optional
Restriction sites	Required	Optional
Statistics information	Little	Comprehensive
Running time (test data 10 million)	131 min	23 min (8 threads)

**Table 6:** Statistics of strand and end distributions.

	s, s	s, t	t, s	t, t
+, +	728,201	25,161,226	25,017,890	274,145
+, −	25,307,505	440,538	566,742	24,765,566
−, +	25,317,128	561,172	442,956	24,739,773
−, −	872,999	25,152,010	24,965,493	171,954

“+” and “−” represent the orientation of alignment, “s” indicates reads located in the 5′ end of the restriction fragment, and “t” means reads located in the 3′ end of the restriction fragment.

## Supplementary Materials

The following are available online at https://www.mdpi.com/2073-4425/11/3/289/s1: Supplementary 1: Table S1: The cosine similarity and PCC between the DLO Hi-C Tool and published data, Supplementary 2: The HTML result report of an example, Supplementary 3: The detailed introduction to the HTML results, Supplementary 4: Detailed introduction of the DLO Hi-C Tool (Python version).

## Abbreviations

3C	Chromosome Conformation Capture
3D	Three-Dimensional
BWA	Burrows-Wheeler Alignment
ChIA-PET	Chromatin Interaction Analysis by Paired-End Tag Sequencing
DLO Hi-C	Digestion-Ligation-Only Hi-C
HTML	Hyper Text Markup Language
PAGE	Polyacrylamide Gel Electrophoresis
PCR	Polymerase Chain Reaction
SNP	Single Nucleotide Polymorphism
TADs	Topologically Associating Domains
