# Supplementary material for: The DLO Hi-C Tool for Digestion-Ligation-Only Hi-C Chromosome Conformation Capture Data Analysis
Source: Genes (Basel). 2020 Mar 10;11(3):289. doi: 10.3390/genes11030289 (PMC7140825; doi:10.3390/genes11030289)
Supplement: Supplementary file 1 [file genes-11-00289-s001.zip › Supplemantary 1.pdf]

Table S1. The cosine similarity and PCC between DLO Hi-C Tool and published data

	Cosine similarity		Pearson correlation coefficient	
	K562-MseI-1	K562-MseI-2	K562-MseI-1	K562-MseI-2
whole genome	0.99995	0.99996	0.99996	0.99996
chr1	0.99985	0.99985	0.99985	0.99985
chr2	0.99986	0.99987	0.99986	0.99987
chr3	0.99980	0.99980	0.99980	0.99980
chr4	0.99979	0.99980	0.99979	0.99980
chr5	0.99985	0.99986	0.99985	0.99986
chr6	0.99981	0.99981	0.99980	0.99981
chr7	0.99986	0.99987	0.99986	0.99987
chr8	0.99982	0.99983	0.99982	0.99983
chr9	0.99980	0.99979	0.99980	0.99979
chr10	0.99982	0.99983	0.99982	0.99983
chr11	0.99984	0.99985	0.99984	0.99985
chr12	0.99986	0.99986	0.99986	0.99986
chr13	0.99983	0.99984	0.99983	0.99984
chr14	0.99983	0.99984	0.99983	0.99985
chr15	0.99986	0.99985	0.99985	0.99986
chr16	0.99980	0.99980	0.99980	0.99980
chr17	0.99985	0.99985	0.99985	0.99985
chr18	0.99985	0.99985	0.99985	0.99985
chr19	0.99976	0.99977	0.99976	0.99977
chr20	0.99982	0.99983	0.99982	0.99982
chr21	0.99987	0.99987	0.99987	0.99987
chr22	0.99969	0.99964	0.99969	0.99964
chrX	0.99986	0.99986	0.99986	0.99986
