# Supplementary material for: The DLO Hi-C Tool for Digestion-Ligation-Only Hi-C Chromosome Conformation Capture Data Analysis
Source: Genes (Basel). 2020 Mar 10;11(3):289. doi: 10.3390/genes11030289 (PMC7140825; doi:10.3390/genes11030289)
Supplement: Supplementary file 1 [file genes-11-00289-s001.zip › Supplementary 2.html]

DLO-HiC Analyse Tool


DLO-HiC

- Running information
  - Configure information
- Linker Filtering
  - Basic statistics
  - Base distribution in adapter
    detection
  - Linker score distribution
  - Tag length distribution
- Alignment
  - Basic statistics
- Noise Reduction
  - Basic statistics
  - Orientation-Position statistics
  - Annotation statistics
  - Interaction distance distribution
- Matrix report
  - Basic
  - Heatmap

2020-2-24 16:59:39

# Running information

## **Configure information**

## | | | | | | --- | --- | --- | --- | | Input file: | DLO-HIC-Lane2-1\_combined\_R1.fastq.gz | Min Linker length: | 32 | | Output folder: | . | Max reads length: | 20 | | Output prefix: | K562-MseI-rep3-NoIter | Match score: | 1 | | Genome file: | /public/home/hjiang/data/fasta/Hg19-HP.fa | MisMatch score: | -1 | | Genome index prefix: | /public/home/hjiang/data/index/Hg19-HP | InDel score: | -1 | | HalfLinkerA: | GTCGGAGAACCAGTAGCT | Resolution: | 1000000 | | HalfLinkerB: | | Thread: | 16 | | Restriction: | T^TAA | | |

---

# Linker Filter

## **Basic statistics**

## | | | | | --- | --- | --- | | Adapter Sequence: | GATCGGAAGAGCACACGTCTGAACTCCAGTCACCGATGTATCCCGTATGCCGTCTTCTGCTTGAAAAAAAAA | | | Total reads: | 262,150,931 | 100.00% | | AA: | 231,638,584 | 88.36% | | Ambiguous: | 30,512,347 | 11.64% | | Valid pair: | 231,617,696 | 88.35% | | Output folder: | ./01.PreProcess | |

## Base distribution in adapter detection

## 

## Linker alignment score distribution

## 

## Tag length distribution

## 

---

# Alignment

## **Basic statistics**

- ### Linker AA: GTCGGAGAACCAGTAGCTAGCTACTGGTTCTCCGAC

  ## | | | | | | | | --- | --- | --- | --- | --- | --- | | Item | Number | Percentage | Item | Number | Percentage | | Fastq file R1: | 231,617,696 | 100.00% | Fastq file R2: | 231,617,696 | 100.00% | | Unique map R1: | 178,978,021 | 77.27% | Unique map R2: | 176,490,219 | 76.20% | | Multi map R1: | 41,097,271 | 17.74% | Multi map R2: | 40,836,508 | 17.63% | | Unmap R1: | 11,542,403 | 4.98% | Unmap R2: | 14,290,969 | 6.17% | | Merge: | 141,840,306 | 61.24% | | | | | Output folder: | ./02.Alignment | | | | |

## ---

# Noise Reduction

## **Basic statistics**

Input: 141,840,306

## | | | | | --- | --- | --- | | Item | Number | Percentage | | Self-Ligation: | 374,901 | 0.26% || ReLigation: | 941,852 | 0.66% | | Duplicate: | 11,320,066 | 7.98% | | Clean data: | 129,203,487 | 91.09% | | Intra-chrom: | 107,014,197 | 82.83% || Inter-chrom: | 22,189,290 | 17.17% | | Short range: | 25,112,008 | 23.47% || Long range: | 81,902,189 | 76.53% |

## Orientation-Position statistics

'+' and '-' represent the orientation of alignment, 's' means reads located in the 5' end of restriction
fragment and 't' means reads located in the 3' end of restriction fragment

## | | | | | | | --- | --- | --- | --- | --- | | | s,s | s,t | t,s | t,t | | +,+ | 378,948 | 15,772,559 | 15,987,520 | 112,356 | | +,- | 16,090,609 | 223,671 | 272,802 | 15,782,325 | | -,+ | 16,081,437 | 271,439 | 224,411 | 15,766,409 | | -,- | 442,222 | 15,760,892 | 15,959,727 | 76,160 |

## Annotation statistics

## Interaction distance distribution

## 

## 

## 

## 

# Matrix report

### Basic statistics

## 

## Interaction heatmap

- #### Resolution 1,000,000 bp

  ##

### Running time

## | | | | --- | --- | | Item | Value(h/m/s) | | Start time: | Mon Feb 24 13:28:13 CST 2020 | | Linker filtering: | 0H54M20S | | Mapping: | 1H44M36S | | Noise Reduction: | 0H36M41S | | Create matrix: | 0H15M22S | | Total: | 3H31M0S |

1. Please use Chrome, Firefox or Safari for better browsing experience.

2. Detailed explanation can be found at
https://github.com/GuoliangLi-HZAU/DHat.

dd
