# Supplementary material for: The DLO Hi-C Tool for Digestion-Ligation-Only Hi-C Chromosome Conformation Capture Data Analysis
Source: Genes (Basel). 2020 Mar 10;11(3):289. doi: 10.3390/genes11030289 (PMC7140825; doi:10.3390/genes11030289)
Supplement: Supplementary file 1 [file genes-11-00289-s001.zip › Supplementary 3.pdf]

DLO Hi-C TOOL RESULTS with an example data

Here, we gave an example of DLO Hi-C data analysis with DLO Hi-C Tool. The example data is the THP-1-MseI-1 data set. It is worth mentioning that all these results are included in the HTML report generated by DLO Hi-C Tool.

Preparation for the data analysis
Installation of the program: “java –jar Dhat.jar install”
Required supporting programs: bwa (repuired), mafft (optional), python (required)
Required data sets: fastq or fastq.gz file
Required supporting data: reference genome file

RUNNING INFORMATION

We provided a table about the configuration information during running, which contains all the important parameters set by the user or by default (for example, input file, output folder, output prefix, reference genome file, reference genome index prefix, the sequences of half linkerA and half linkerB, and so on).

Input file:	DLO-HIC-Lane2-1_combined_R1.fastq.gz	Min Linker length:	33
Output folder:	.	Max reads length	20
Output prefix:	K562-MseI-rep3-NoIter	Match score:	1
Genome file:	/public/home/hjiang/data/fasta/Hg19-HP.fa	MisMatch score:	-1
Genome index prefix:	/public/home/hjiang/data/index/Hg19-HP	InDel score:	-1
HalfLinkerA:	GTCGGAGAACCAGTAGCT	Resolution:	1000000
HalfLinkerB:		Thread:	16
Restriction:	T^TAA		

LINKER FILTER RESULTS

- **Basic Statistics**

In the section of Basic Statistics, a table will show all statistical results for linker filtering. Adapter sequence is provided by users or set to automatic. When the parameter “adapter sequence” is ‘auto’, it means that users lacked the information of linker sequence and the software will detect the linker sequence automatically.

When the program runs, it will report a table with the numbers and percent of the total reads, reads contains different combination of half-linkers, and ‘ambiguouts’ reads (we can't determine whether this read contains linker, or can’t define the linker type). There are 4 combinations (AA, BB, AB and BA) of half-linkers in DLO Hi-C and 1 combination (AA) of half-linkers in *in situ* DLO Hi-C library. Usually in a normal library, a high fraction of reads is expected to contain linkers, “Valid pair” represent the reads pair that will used to alignment. In the end, all linker filtering results had stored in the output folder.

Adapter Sequence:	GATCGGAAGAGCACACGTCTGAACTCCAGTCACCGATGTATCCCGT ATGCCGTCTTCTGCTTGAAAAAAAAA	
Total reads:	262,150,931	100.00%
AA:	231,638,584	88.36%
Ambiguous:	30,512,347	11.64%
Valid pair:	231,617,696	88.35%
Output folder:	./01.PreProcess	

- **Base distribution in adapter detection**

When users use auto adapter detection function, DLO Hi-C Tool will generate the result for adapter detection. By default, it will take the first 200 reads and do MSA (multiple sequence alignment), this figure showed the base distribution in MSA. Different colors represent different bases, and purple means gap. Based on the design of the experiment, the adapter sequence is always in certain position of the reads and just one type of adapter is in one DLO Hi-C experiment. If one base appears in a large proportion of the reads in a certain position and there are multiple positions in a continuous region, this continuous region is very likely to be the adapter position.

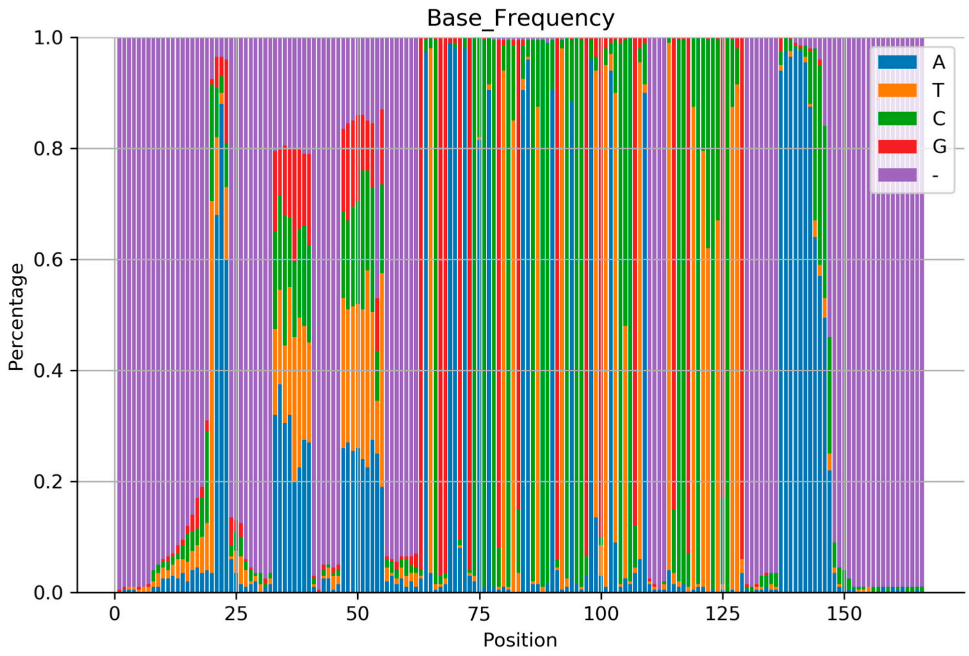

- **Linker alignment score distribution**

To assess the result of linker alignment, the report gave a figure of linker alignment score distribution. By default, match score will be set as 1, and mismatch score and InDel score are set to -1. Generally, DLO Hi-C DNA fragment contains about 40 bp linker sequences. If most of the reads have high linker mapping scores, it means that the data quality of this experiment is good. Actually, for a qualified DLO Hi-C experimental data, its reads linker mapping scores will have a significant peak at the

score >35. If the peak is at 10 or below, it means that this data does not include the linker. Users need to consider that the linker sequence is set incorrectly.

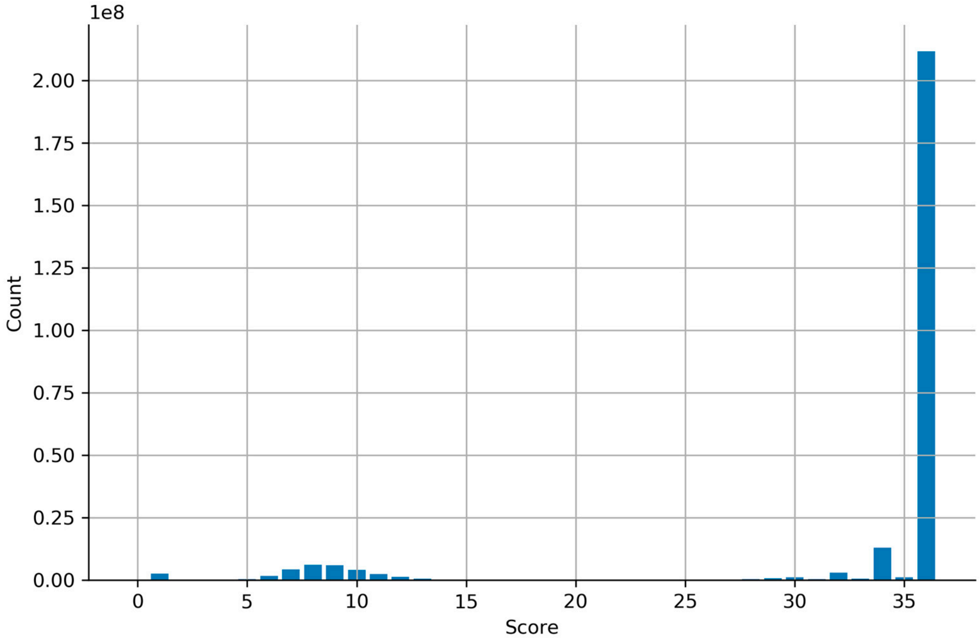

- **Tag length distribution**

According to the experiment design of DLO Hi-C protocol, DLO Hi-C DNA fragment contains two interacting genomic DNAs, 40 bp linker sequences and adapter bases. In our pipeline, we called these two interacting genomic DNAs as R1 and R2 tags (different from R1 and R2 reads of paired-end sequencing). This figure showed R1 and R2 tag length distribution, and we usually expect to see that most tags are 21 or 22 base pairs.

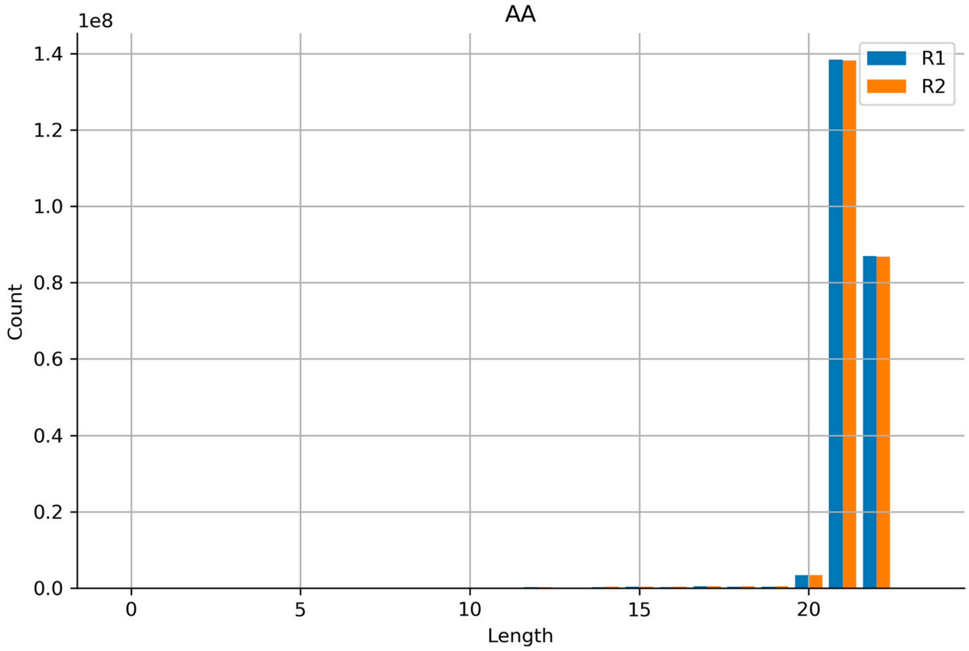

ALIGNMENT

- **Basic Statistics**

The mapping statistics is available in this section. Usually, a high fraction of reads is expected to be uniquely aligned on the genome. A high level of multiple mapping and un-mapping is associated with a low quality experiment. When both R1 tag and R2 tag on one fragment are aligned on the genome, DLO Hi-C tool merged them together and re-constructed pair information. The *02.Alignment* folder is used to store the alignment products.

Linker AA: GTCGGAGAACCAGTAGCTAGCTACTGGTTCTCCGAC

Item	Number	Percentage	Item	Number	Percentage
Fastq file R1:	231,617,696	100.00%	Fastq file R2:	231,617,696	100.00%
Unique map R1:	178,978,021	77.27%	Unique map R2:	176,490,219	76.20%
Multi map R1:	41,097,271	17.74%	Multi map R2:	40,836,508	17.63%
Unmap R1:	11,542,403	4.98%	Unmap R2:	14,290,969	6.17%
Merge:	141,840,306	61.24%			
Output folder:	./02.Alignment				

NOISE REDUCE

- **Basic Statistics**

Statistics about read pairs filtering is available in this section. We classified some of the paired-end reads as invalid interactions, such as self-ligation and re-ligation reads. A high level of self-ligation or re-ligation read pairs is associated with a low quality DLO Hi-C experiment, which may be related problems during the experiment.

In addition, due to polymerase chain reaction (PCR) amplification, the same reads may be sequenced many times. A high level of duplication shows a poor molecular complexity and a potential PCR bias.

Then, one important index is the fraction of intra and inter-chromosomal interactions, as well as the proportion of long range (> 5kb for four-base enzyme, and > 20kb for six-base enzyme) and short range (<5kb for four-base enzyme, and <20kb for six-base enzyme) intra-chromosomal interactions.

Input: 141,840,306

Item	Number	Percentage
Self-Ligation:	374,901	0.26%
ReLigation:	941,852	0.66%
Duplicate:	11,320,066	7.98%
Clean data:	129,203,487	91.09%
Intra-chrom:	107,014,197	82.83%
Inter-chrom:	22,189,290	17.17%
Short range:	25,112,008	23.47%
Long range:	81,902,189	76.53%

- **Orientation-Position Statistics**

One additional quality control is the orientation-position statistics. In this table, '+' and '-' represent the orientation of alignment, 's' means reads located in the 5' end of restriction fragment and 't' means reads located in the 3' end of restriction fragment. From the experimental design of Hi-C, it's clearly that an ideal result should be that almost all reads belong to 8(+,-/s,s; -,+/s,s; +,+/s,t; -,-/s,t; +,+/t,s; -,-/t,s; +,-/t,t; -,+/t,t) of 16 cases.

	s,s	s,t	t,s	t,t
+,+	378,948	15,772,559	15,987,520	112,356
+,-	16,090,609	223,671	272,802	15,782,325
-,+	16,081,437	271,439	224,411	15,766,409
-,-	442,222	15,760,892	15,959,727	76,160

- **Interaction distance distribution**

The content of this section is the distance distribution for all intra chromosome interaction. Here X label represents the distance of reads pairs, Y label represents the count of the corresponding distance (take count log10). In X label, the figure between first brackets indicates the minimum unit of distance, the figure between second brackets means take distance log2, the figure between square brackets means the range of distance. In general, X and Y values have almost linear correlation in short distance range.

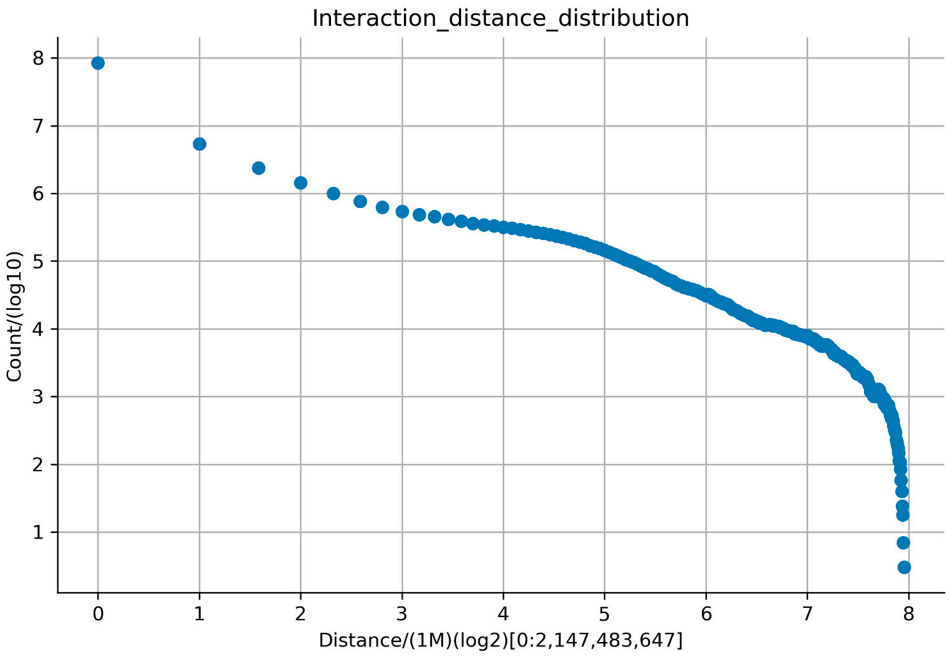

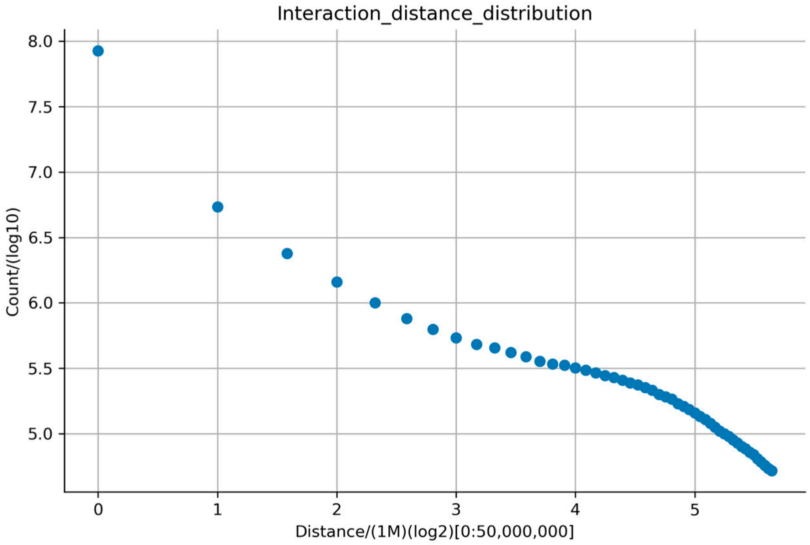
Interaction_distance_distribution
Count/(log10)
8.0
7.5
7.0
6.5
6.0
5.5
5.0
0
1
2
3
4
5
Distance/(1M)(log2)[0:50,000,000]

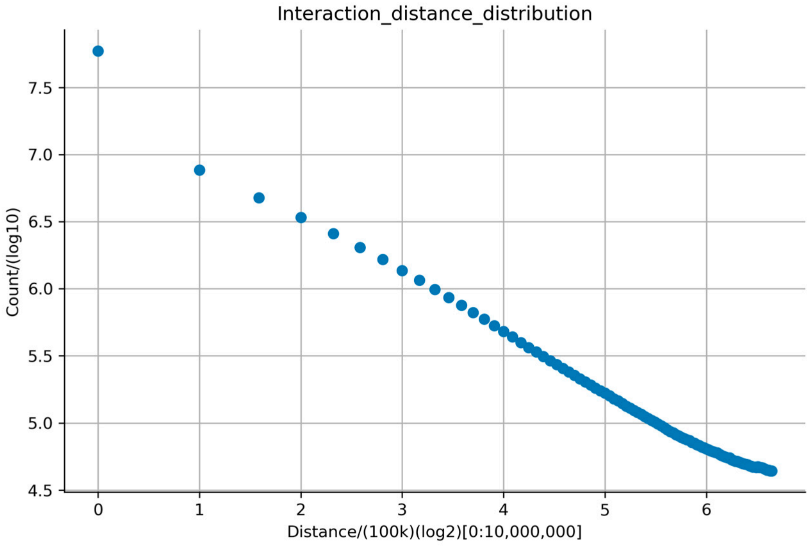
Interaction_distance_distribution
Count/(log10)
7.5
7.0
6.5
6.0
5.5
5.0
4.5
0
1
2
3
4
5
6
Distance/(100k)(log2)[0:10,000,000]

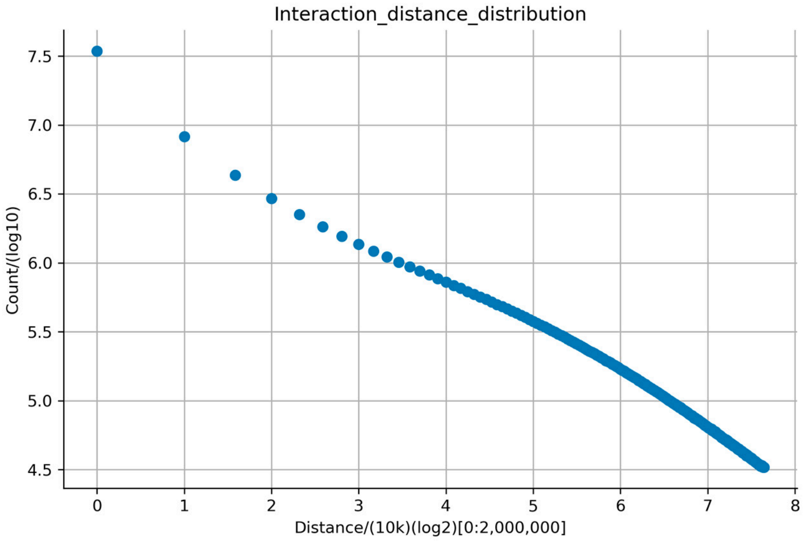
Interaction_distance_distribution
Count/(log10)
7.5
7.0
6.5
6.0
5.5
5.0
4.5
0
1
2
3
4
5
6
7
8
Distance/(10k)(log2)[0:2,000,000]

MATRIX REPORT

- **Interaction Heatmap**

Actually, a heatmap is probably the most direct and widely used visualization method for a Hi-C contact matrix. Here we provided heat maps constructed for the full genome. Heatmaps constructed for individual chromosomes are then available in the CreateMatrix folder.

Resolution 1,000,000 bp

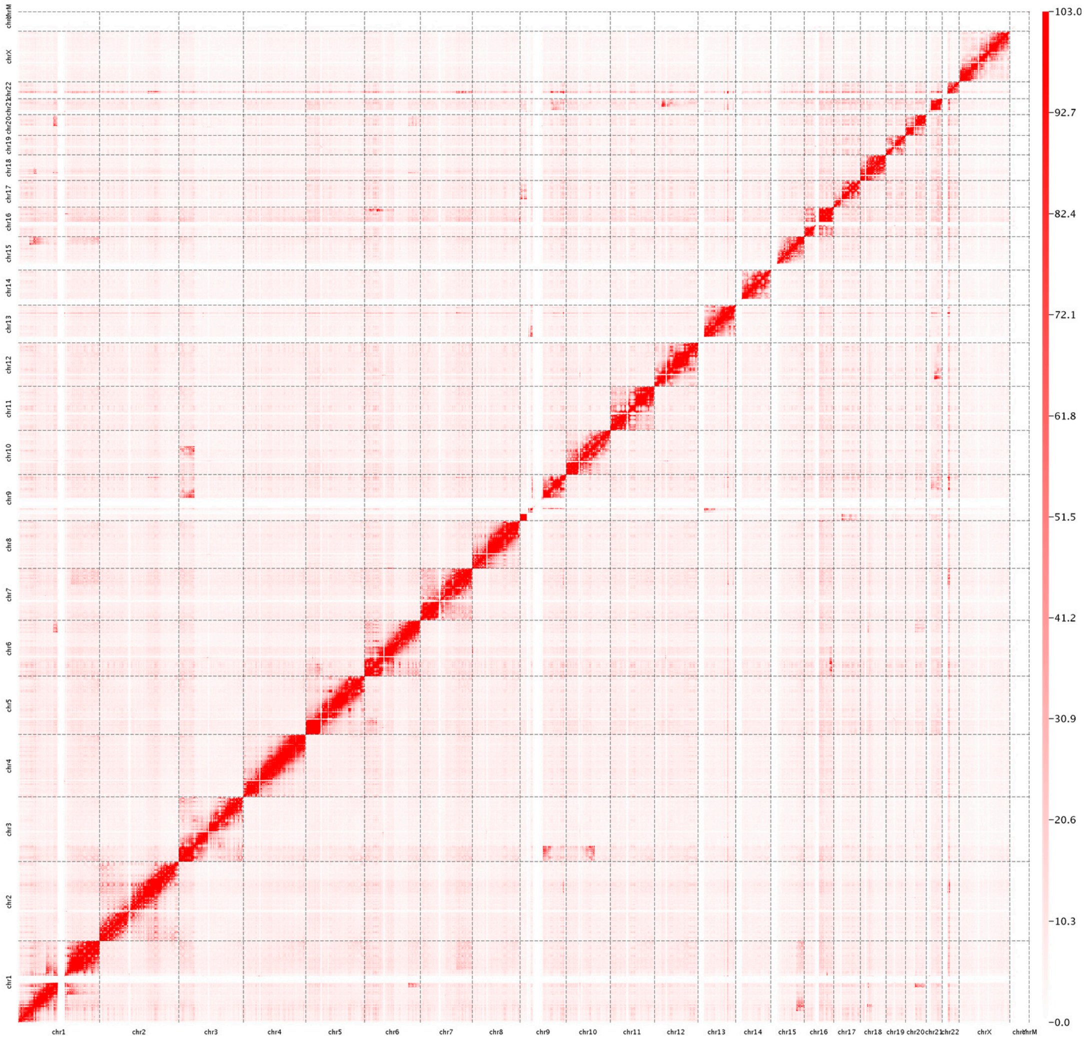

- **Running Time**

At last, the software will provide a table of running time information. User can get the start time, specific running time for each step, and total running time from this table. Generally speaking, alignment step is the most time-consuming of all steps. Increasing the number of threads can effectively reduce running time.

Item	Value(h/m/s)
Start time:	Mon Feb 24 13:28:13 CST 2020
Linker filtering:	0H54M20S
Mapping:	1H44M36S
Noise Reduce:	0H36M41S
Create matrix:	0H15M22S
Total:	3H31M0S
